# Cure of Flat-Foot by Message

**Published:** 1890-03-08

**Authors:** 


### CURE OF FLAT-FOOT BY MASSAGE.

The origin of this deformity is uncertain, and the treatment has hitherto been far from satisfactory, but Dr. Landerer, of Leipzig, reasoning from the indisputable fact that weakness of the muscles, though not the only factor, plays an important part in the production of malformations of the foot, has tried the effect of treatment by massage with a view to strengthening them. The muscles on which he operates are the tibialis porticus, those of the calf and of the sole. His first case was a young man, aged 19, who had suffered so painfully for six years that the head of the fourth metatarsal had been excised, and amputation of the foot had actually been recommended. After two months' persevering massage the arch was to a great extent restored, and the patient experienced no pain in walking. Seven years have now elapsed and he can dance and walk for hours without distress. In two other cases Dr. Landerer has been equally successful, but in an even shorter time.

